# Layer-by-layer coating strategy to functionalize the magnetic nanoparticles for their multi-functionalization

**DOI:** 10.1186/s11671-025-04250-6

**Published:** 2025-05-02

**Authors:** Jing Liu, Ye Chen, Hongjie Huang, Feixiong Chen

**Affiliations:** 1 Department of Research, Shanghai University of Medicine and Health Sciences Affliated Zhoupu Hospital, The College of Medical Technology, Shanghai University of Medicine and Health Sciences, Shanghai, 201318 China; 2 Huangyan District Center for Disease Control and Prevention, Taizhou, 318020 Zhejiang China; 3 Department of Sports Medicine, Peking University Third Hospital; Institute of Sports Medicine of Peking University; Beijing Key Laboratory of Sports Injuries; Engineering Research Center of Sports Trauma Treatment Technology and Devices, Ministry of Education, Beijing, China; 4 Centre for BioNano Interactions, School of Chemistry and Chemical Biology, University College Dublin, Dublin, Dublin 4, Ireland; 5 Disease Networks Research Unit, Faculty of Biochemistry and Molecular Medicine, University of Oulu, 90014 Oulu, Finland

**Keywords:** Magnetic nanoparticles, Electroactive molecules, Layer-by-layer (LbL) technique, Electrochemical sensing, Bio-nano interaction

## Abstract

Magnetic nanoparticles (MNPs) hold significant potential for a wide range of applications, however, surface modification or bio-conjugation of MNPs often leads to their aggregation and instability. To address this, we proposed a facile method using a layer-by-layer (LbL) coating technique with polyallylamine hydrochloride (PAH) and poly(styrene sulfonic acid) sodium salt (PSS), so as to maintain the dispersion stability and functionality of MNPs. This method enabled us to develop the powerful MNPs towards to their use in the electrochemical biosensor, by combining both the redox probes (ferrocene (Fc), anthraquinone (AQ), or monocarboxymethylene blue (MB)) and bio-probes (IgG). The redox molecules were effectively anchored to the MNPs under the organic solvents, while such functionalized MNPs surface were subsequently protected by the LbL coating process prior to dispersing in high ionic strength solutions (e.g. Phosphate-buffered saline). And the out-layer of polyelectrolyte shell allowed biomolecules to attach to the MNP surface without chemical cross-linking. Our results demonstrated that the TEM size of MNPs@Fc, MNP@AQ and MNP@MB after LbL coating were characterized as 11.0 ± 2.0 nm, 10.5 ± 2.1 nm and 12.4 ± 2.2 nm and these developed redox MNPs of MNPs@Fc, MNPs@AQ and MNPs@MB were characterized by square wave voltammetry (SWV) with their redox intensity of 0.64 ± 0.10 µA, 23.25 ± 0.73 µA and 0.48 ± 0.13 µA, respectively. In addition, the binding efficiency of adsorption between the MNPs and IgG was up to 78%, evidenced by SDS-PAGE gel analysis. This facile method offered a versatile and effective way to functionalize MNPs, combining redox and biological properties for potential applications in disease diagnosis and point-of-care diagnostics.

**Supplementary Information:**

The online version contains supplementary material available at 10.1186/s11671-025-04250-6.

## Introduction

In the recent years, magnetic nanoparticles (MNPs) have emerged as a promising nanomaterials, due to their ease of preparation, high surface-to-volume ratio, and bio-compatibility [1]. Extensive research has focused on the development of MNPs for various applications, including bio-separation [2–5], bio-sensing [6], and drug delivery [7]. Notably, the desirable surface modification of MNPs is essential for effectively utilizing their physical properties. However, newly synthesized bare MNPs tend to rapidly agglomerate or undergo surface oxidation, significantly limiting their applications [8, 9]. For instance, the interface of bare MNPs can be significantly altered when exposed to biofluids, often resulting in the release of metal ions, such as Fe^2+^ ions [10]. Additionally, owing to their lack of enough surface charge density, bare MNPs are unable to generate sufficient electrostatic repulsion, increasing their risk of aggregation [11]. This leads to the loss of their physical properties and a decrease of colloidal stability. Therefore, surface modification strategies are crucial to stabilize MNPs in complex media like physiological fluids, enabling their use in a wide range of applications.

Colloidal stability and desirable functionality of MNPs are critically dependent on their surface modifications, surface charges, surface groups and surface chemistry. To achieve this, several strategies have been developed for the MNP surface modification, such as amination [12], PEGylation [12, 13], and bio-conjugation [14, 15]. However, chemical modifications such as carbodiimide crosslinker chemistry, maleimide chemistry, and click chemistry involve several steps, including chemical group activation, chemical modification and covalent binding [3, 16]. These modification processes can alter the MNP surface groups and reduce their surface charge density, potentially increasing the risk of aggregation under a high ionic strength buffer [17], like phosphate buffered saline (PBS). In addition, some of surface groups anchored at MNP surface might also exhibit their strong hydrophobicity, directly causing the MNP aggregation, even in aqueous buffer [3, 18, 19]. Therefore, it is necessary to develop an approach for effective surface modification for their multi-functionality and against the aggregation risk of MNPs during the surface functionalization process.

In contrast, layer-by-layer (LbL) assembly technique is a promising and facile strategy to form highly charged surface coatings around MNPs, owing to their simplicity, versatility and nanometer-scale control [20–22]. On one hand, the LbL coating process can be driven by electrostatic interactions between negatively charged and positively charged polyelectrolyte layers. Without the needs of chemical modifications, this method allows for grafting sufficient surface charge density onto the MNP surface, thereby preventing these MNPs from aggregation [23, 24]. On the other hand, surface functionalization of MNPs for various applications typically involves using different molecules, such as electroactive molecules [16], fluorescent markers [25], or drugs [26]. Given that majority of these conjugated molecules contain hydrophobic groups and have low solubility in aqueous solutions, organic solvents such as dimethyl sulfoxide (DMSO), acetone, and ethanol are more suitable for their use in the chemical modification process for MNPs, than that of aqueous buffer. Thus, in order to transfer these surface-modified MNPs from organic solvents to aqueous solutions, the LbL coating process helps protect the hydrophobic surface of MNPs from aggregation and ensures their compatibility in aqueous dispersion [27, 28], prior to their bio-application. Additionally, benefiting from the LbL technique, the outer polyelectrolyte layer of MNPs not only stabilize the MNP dispersion but also provide a suitable and desirable adsorption interface for the attachment of biomolecules such as antibody, avoiding the dependence of further chemical crosslinking [29, 30].

To address the issue of MNP aggregation caused by both molecules conjugation and bio-immobilization, this work aimed to report a facile strategy of LbL coating process for surface functionalization of MNPs, in terms of their stabilization and multi-functionality.

**Fig. 1:**
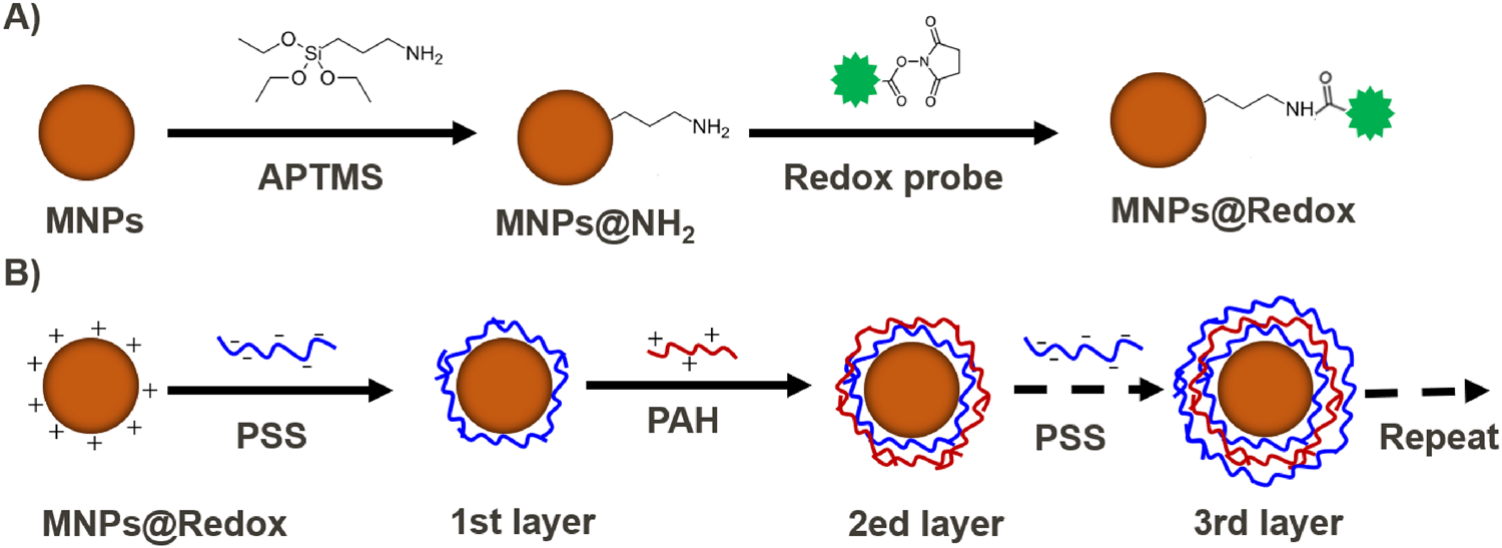
Surface functionalization of MNPs by using layer-by-layer (LbL) strategy. **A** Redox conjugation and amination of the magnetic nanoparticles (MNPs) by APTMS and redox probe, **B** Layer-by-layer coating method to stabilize the redox MNPs

As the multifunctionalities of MNPs proposed in our work enable the efficient magnetic separation, sensitive redox readout and selective bio-capture [31], MNP-derived electrochemical sensors offer the immense possibility of diagnosing and detecting disease biomarkers more efficiently [6]. Li et al. [32] and Feng et al. [33] described different methods for combining redox reactions and antibodies on MNP surfaces, allowing for their unique functionalities like bio-capture or bio-sensing. As described in Fig. 1A, our strategy was to covalently conjugate these redox probes onto the MNP surface, following the synthesis and amination of MNPs. Given that the majority of redox probes, such as ferrocene (Fc), methylene blue (MB), and anthraquinone (AQ), are hydrophobic in nature [34–37], the initial redox functionalized MNPs were prepared and stored in the organic solvent of DMSO. Then, via the LbL coating process in Fig. 1B, these polyelectrolyte layers were coated onto the hydrophobic surface of redox MNPs to keep their aqueous dispersion stability. In addition to their redox property, the outer polyelectrolyte layer of MNPs provided a suitable adsorption interface for the attachment of antibodies, to form a biomolecule surface for specific bio-capture.

As the novel aspect of our strategy, the utilization of organic solvents for MNP synthesis and chemical modifications made our strategy to be a versatile and common approach, because it can be compatible with various surface groups, surface charge densities, and surface hydrophobicity. Following the LbL coating process, our approach facilitated the physical adsorption of biomolecules onto the MNP surface, eliminating the needs of further chemical crosslinking. Beyond the redox moieties and antibodies, our strategy can also be extended to meet needs of different surface functionalization of MNPs, broadening their potential applications.

## Experimental

### Materials

Anthraquinone-2-carboxylic acid n-succinimidyl ester (AQ) and monocarboxymethylene blue n-succinimidyl ester (MB) were obtained from EMP Biotech GmbH (Germany), while ferrocenecarboxylic acid n-succinimidyl ester (Fc) was purchased from Tokyo Chemical Industry UK Ltd. (Japan). Poly(styrene sulfonic acid) sodium salt (PSS, Mw70,000) and polyallylamine hydrochloride (PAH, Mw 50,000) were purchased from Alfa Aesar and Sigma Aldrich, respectively. Additional chemical reagents, such as N-hydroxysuccinimide (NHS), N-(3-dimethylaminopropyl)-N’-ethylcarbodiimide hydrochloride (EDC), hydrochloric acid (HCl), iron dichloride tetrahydrate (FeCl_2_ 4H_2_O), iron (III) chloride hexahydrate (FeCl_3_.6H_2_O), 4-(2-Hydroxyethyl)piperazine-1-ethane-sulfonic acid (HEPES), phosphate buffered saline (PBS), immunoglobulin G (IgG), and (3-aminopropyl)trimethoxysilane (APTMS), were all purchased from Sigma‒Aldrich. LS Columns used for magnetic separation were obtained from Miltenyi Biotec (Germany). All chemicals were used as received without any further purification.

### Synthesis of ***γ***-Fe_2_O_3_ nanoparticles

As described in Chen et al. [12], iron nanoparticles (*γ*-Fe_2_O_3_) were synthesized by the co-precipitation method. Briefly, FeCl_3_.6H_2_O (0.030 mol) in 350 mL of Milli-Q deionized (DI) water and FeCl_2_ 4H_2_O (0.015 mol) in 17.0 mL of hydrochloric acid (HCl, 1.5 mol L^−1^) were vigorously mixed under mechanical stirring. Then, 30.0 mL of 35% ammonia solution was added to form the iron oxide of Fe_3_O_4_ nanoparticles. After washing three times in DI water, these nanoparticles were dispersed in 25 mL of nitric acid (2.0 mol L^−1^) for 15 min to partly oxidize the nanoparticle surface. Finally, these nanoparticles were mixed with 60.0 mL of iron nitrate (0.33 mol L^−1^) for 30 min to fully oxidize these nanoparticles to *γ*-Fe_2_O_3_ nanoparticles, as the tested MNPs used in this study.

### Amination and redox conjugation of MNPs

5.0 mL of MNPs at a concentration of 38.5 mg mL^−1^ was mixed with 10.0 mL of ethanol, and 0.36 mL of APTMS (97%) was added for incubation overnight. With the additional glycerol of 10.0 mL, the mixture was allowed to react via amination at 95 °C and 45 mbar for 2 h under a rotary evaporator [12, 38]. After washing with large amount of acetone, the MNPs@NH_2_ were collected in 5.0 mL of DI water. By the Schenk line, we transferred the MNPs@NH_2_ from the aqueous to the dimethyl sulfoxide (DMSO). Concentration of MNPs was characterized by the colorimetric titration described in 2.5.

In terms of redox conjugation, 0.22 mL of MNPs@NH_2_ (22.5 mg mL^−1^) and 0.015 mmol of redox probes (Fc, AQ, MB) was mixed together for overnight incubation at 60 °C and 550 rpm, where the NHS-ester based redox probes might directly react with the amine group (–NH_2_) at MNPs. By this procedure, we prepared the MNPs@Fc, MNPs@AQ, and MNPs@MB. The ratio of Fc probe per nm^2^ was approximately 10 for the reaction process. Finally, these MNPs@Fc were magnetically collected and concentrated on an LS column (130-042-401, Miltenyi Biotec), to remove the free redox probes and redispersed in DMSO.

### Layer-by-layer (LbL) coating strategy

The LbL method was applied to stabilize the redox MNPs or MNPs@NH_2_ in aqueous solution. Briefly, the initial MNPs@Fc rapidly diluted in 10.0 mL DI water (1.0 mg mL^−1^) was timely dispersed in the 10.0 mL of PSS solution (10.0 mg mL^−1^) for 5 min sonication. Second, MNPs@Fc@PSS with the first layer were collected by centrifugation at 20,000 RPM for 15 min and redispersed in 200.0 µL of DI water. Third, 200.0 µL of PAH (10.0 mg mL^−1^), as a positively charged polymer, was also added to the MNP@Fc@PSS for the second layer coating. The MNPs@Fc@PSS@PAH were further collected by centrifugation at 20,000 RPM for 10 min and redispersed in 200.0 µL of DI water. Again, 200.0 µL of PSS (10.0 mg mL^−1^) was added as the third polymer layer. The MNPs@Fc@PSS@PAH@PSS were collected by centrifugation at 20,000 RPM for 10 min and redispersed in 200.0 µL of DI water. After repeating the coating process for several times, the final MNPs were collected in the DI water with a volume of 100.0 µL (2.0 mg mL^−1^) for characterization and bio-testing.

### Colorimetric titration by UV visible spectra

To quantify the iron oxide concentration of MNPs, 10.0 µL of each sample was mixed with 20.0 µL of DI water and 90.0 µL of hydrochloric acid (37%) for dissolving the iron oxide in the ions of Fe^3+^. Then, 2.9 mL of DI water, 60.0 µL of sodium thiocyanate solution (2.0 mol L^−1^) and 60.0 µL of the above sample were mixed together for colour changes from green to the red. Under the UV absorption at 461 nm, a calibration curve of Fe^3+^ was also recorded to estimate the MNP content in each sample.

To quantify the amine group density of MNPs@NH_2_ via the Ninhydrin testing [12], 200.0 µL of MNPs was mixed with 250.0 µL of ninhydrin solution (20.0 mM) and acetic acid buffer (pH = 5.4) and incubated at 75 °C for 20 min. Supernatant of each sample was collected under UV absorption at 571 nm, where a calibration curve of glycine was also recorded in Fig. S1.

### Characterization and instruments

All the MNPs were suspended in ethanol at a concentration less than 0.1 mg mL^−1^ and deposited on a 200 mesh copper transmission electron microscopy (TEM) grid with a carbon/formvar film (Ted Pella, Inc., Redding, CA, USA; Prod # 01754-F). All the MNPs were dispersed in DI water or in PBS1X buffer for the DLS size measurement via dynamic light scattering (DLS), while the MNPs dispersed in 10 mM HEPES buffer were used for zeta potential measurement. Both of them were performed using a Malvern Zetasizer Nano ZS instrument (Malvern Panalytical Ltd., UK). ALPHA FT-IR Spectrometer (Bruker Optics Inc. MA, USA) was used for the characterization of MNPs before and after surface functionalization by using the attenuated total reflectance (ATR) technique with the 64 scans and a spectral resolution of 4 cm^−1^. An EmStat3 USB-powered potentiostat was selected to characterize the redox signal by square wave voltammetry (SWV) and cyclic voltammetry (CV) with the parameters of a voltage ranging from 0 to 0.7 V and a scanning rate of 100 mV s^−1^ and 40 Hz. Protein characterization was performed using SDS‒PAGE and Coomassie blue staining, where all samples were loaded in an 8% polyacrylamide gel, and gel electrophoresis was performed at 140 mV for 45 min.

## Results and discussion

### Surface characterization of MNPs@NH_2_ and MNPs@Fc

**Fig. 2:**
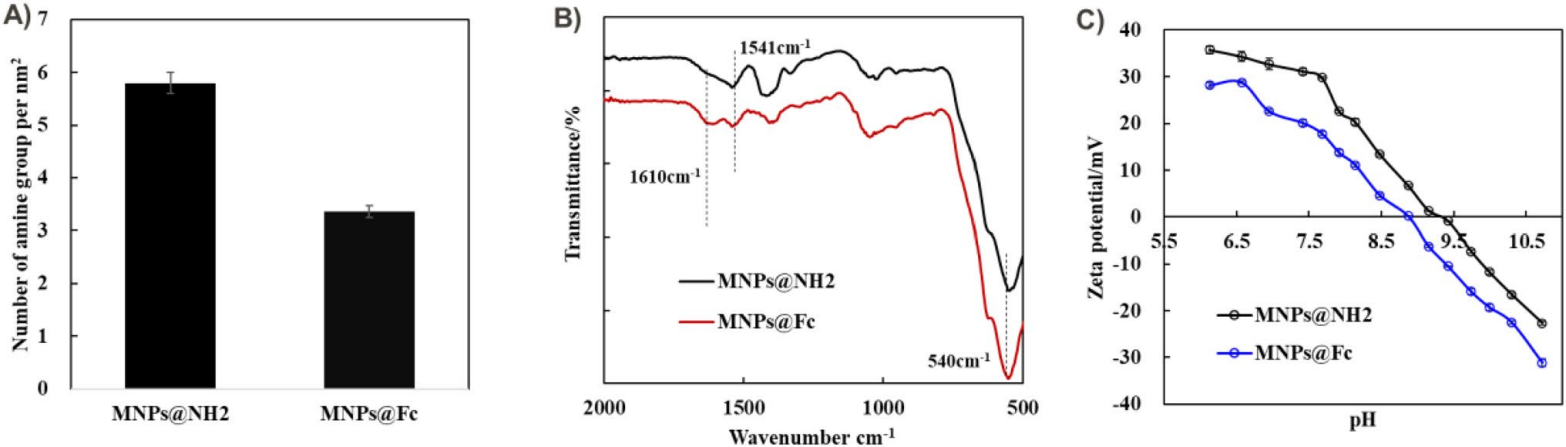
Surface characterization of MNPs@NH_2_ and MNPs@Fc. **A** Amine density of MNPs before and after Fc conjugation; **B** FT-IR spectra of MNPs@NH_2_ and MNPs@Fc. **C** Zeta potential-pH curves of MNPs@NH_2_ and MNPs@Fc under different pH prepared by using 10.0 mM nitric acid and 10.0 mM ammonium hydroxide

First, MNPs were synthesized from iron oxide nanoparticles by the co-precipitation method according to previous methods [39–42]. These MNPs were directly aminated with APTMS to form MNPs@NH_2,_ while the redox probe of Fc-NHS ester were covalently anchored onto the surface of MNPs@NH_2_ through the formation of amide bonding, regards to the preparation of MNPs@Fc. According to the obtained calibration curve in Fig. S1, the amine group density of MNP@NH_2_ and MNP@Fc can be measured by the ninhydrin testing, so as to evaluate the reduction of amine groups caused by Fc conjugation [39]. As showed in Fig. 2A, the amine density of MNP@NH_2_ and MNPs@Fc were reported to be 5.8 ± 0.2 per nm^2^ and 3.4 ± 0.1 per nm^2^, respectively, depending on the calculation methods described in the supplementary materials. It demonstrated that these Fc probes anchored at MNPs surface via the replacement the position of amine groups. According to the depletion of amine density at MNPs surface, Fc conjugation ratio were estimated to be approximately 41%. Secondly, as shown in Fig. 2B, the infrared spectra of MNPs@NH_2_ and MNPs@Fc were also measured by the ATR model to further determine the presence of ferrocene and amine groups. For the sample of MNPs@NH_2_, the FT-IR peak at 1541 cm^−1^ was related to the C-N vibration, reflecting the presence of APTMS at the MNPs surface, while obvious and strong vibration bonding of the Fe–O group was also observed at 592 cm^−1^ [43] Notably, a new FT-IR peak occurred at 1610 cm^−1^ after Fc conjugation, which corresponds to the occurrence of amide bonds due to Fc conjugation. As displayed in the Fig. S2, the zeta potential of MNPs@@NH_2_ exhibited a positive surface charge of 17.8 ± 2.0 mV (in 10 mM HEPES, pH = 7.4), while the zeta potential of MNP@Fc was significantly reduced to −0.9 ± 0.2 mV, owing to the Fc conjugation. Lastly, dependent on their difference in zeta potential, the zeta potential-pH curves of MNPs@NH_2_ and MNP@Fc were recorded in Fig. 2C, indicating that the Fc conjugation caused a shifting of isoelectric point from 9.4 to 8.9. Both of zeta potential measurement and infrared spectroscopy presented the successful surface functionalization of MNPs, such as amination and Fc conjugation.

### LbL coating method for stabilizing redox-active MNPs

To validate the efficiency of LbL process, the negatively charged PSS and positively charged PAH were used as these polyelectrolytes to alternatively graft the surface of MNPs@NH_2_. As demonstrated in Fig. S3A, the MNP@NH_2_ had a positively charged surface, while its surface became a negative zeta potential, after grafting the first polyelectrolytes layer of the polyanions of PSS. Then, by electrostatically adsorbing the polycations of PAH as the second layer, the MNPs’ surface charge became positive again. With the increase of surface coating layers of polyelectrolytes, the MNPs displayed the surface charge reversal from positive to negative, which were aligned with the type of polycations or polyanions during each of layer coating. We demonstrated the feasibility to construct multi-polyelectrolyte layers at the MNPs@NH_2_ surface by LbL coating process. Although the polyelectrolytes can significantly improve the surface charge density of MNPs, the shell structure formed by the coated polyelectrolytes was often random and non-dense, which might lead to an increase in nano-size [24, 44, 45]. Thus, Fig. S3B displayed that the increase of polyelectrolyte layers at MNPs surface resulted in a corresponding increase in the DLS size of MNPs. Clearly, once more than three layer of polyelectrolytes were coated, it seemed the DLS size of MNPs might be stabilized around 160 nm. Notably, the polyelectrolytes shell with coating three layers enabled to stabilize the MNPs in the high-ionic-strength buffer like PBS, as their DLS size (Fig. S3C and D) characterized in both of PBS buffer (pH = 7.4) and DI water were similar without spontaneous disintegration of polyelectrolytes. Thus, at least three coating layers were necessary to construct a polyelectrolyte shell that provided sufficient surface charge density and to ensure the dispersion stability of MNPs.

**Fig. 3:**
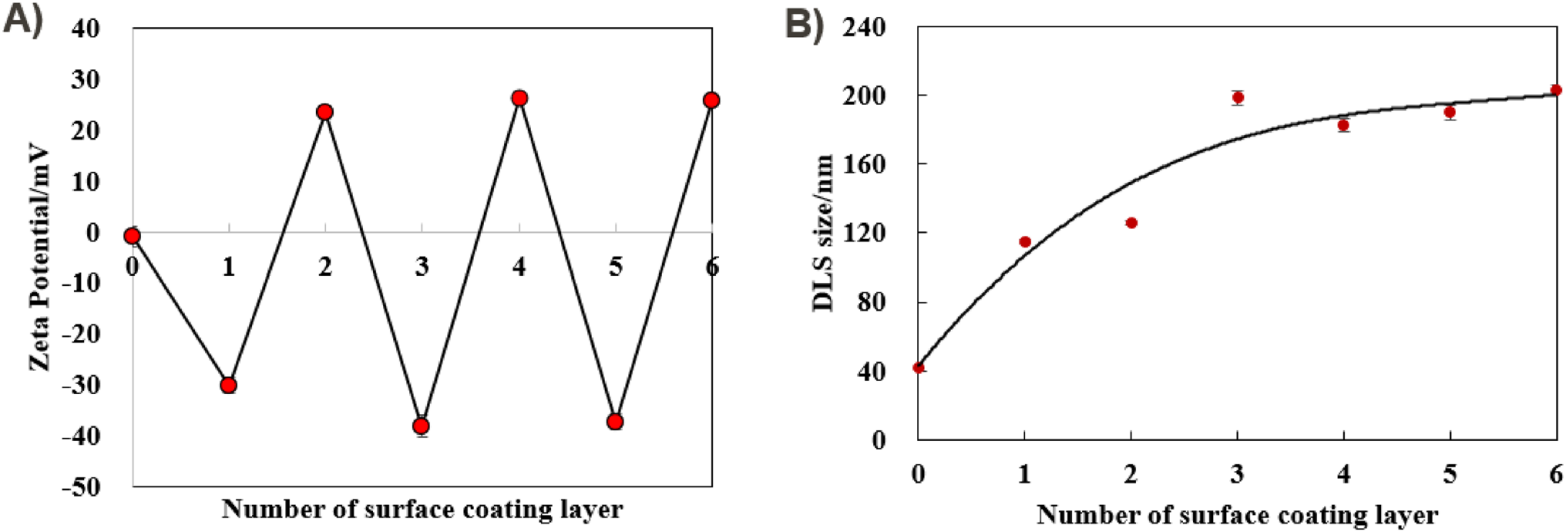
Zeta potential and DLS characterization of MNPs@Fc under the layer-by-layer coating process. **A** Zeta potential of MNPs@Fc coated with different polyelectrolytes layers of PSS or PAH, measured in 10 mM HEPES with pH = 7.4. **B** DLS size of MNPs@Fc after coating different polyelectrolytes layers, measured in DI water

In terms of MNPs@Fc, they exhibited a negative surface charge as measured in HEPES buffer (at pH 7.4). When measured in DI water, their surface charge was found to be 24.4 ± 1.6 mV in Fig. S4A. Herein, we also performed LbL coating process in DI water to graft multi-polyelectrolyte layers of PAH and PSS, for stabilizing these redox MNPs@Fc. Similarly, Fig. 3A also presented a reversal of zeta potential from negative to positive, which can be attributed to the LbL coating process. It also indicated that MNPs@Fc were also alternatively grafted with PSS and PAH. The increase of DLS size for MNPs@Fc were associated to the number of coating polyelectrolyte layers, but their size eventually stabilized around 200 nm, as depicted in Fig. 3B. The difference of DLS sizes between the MNPs@NH_2_ and MNPs@Fc might be attributed to the effects of Fc conjugation on the polyelectrolyte adsorption and their dense of PSS/PAH layer.

**Fig. 4:**
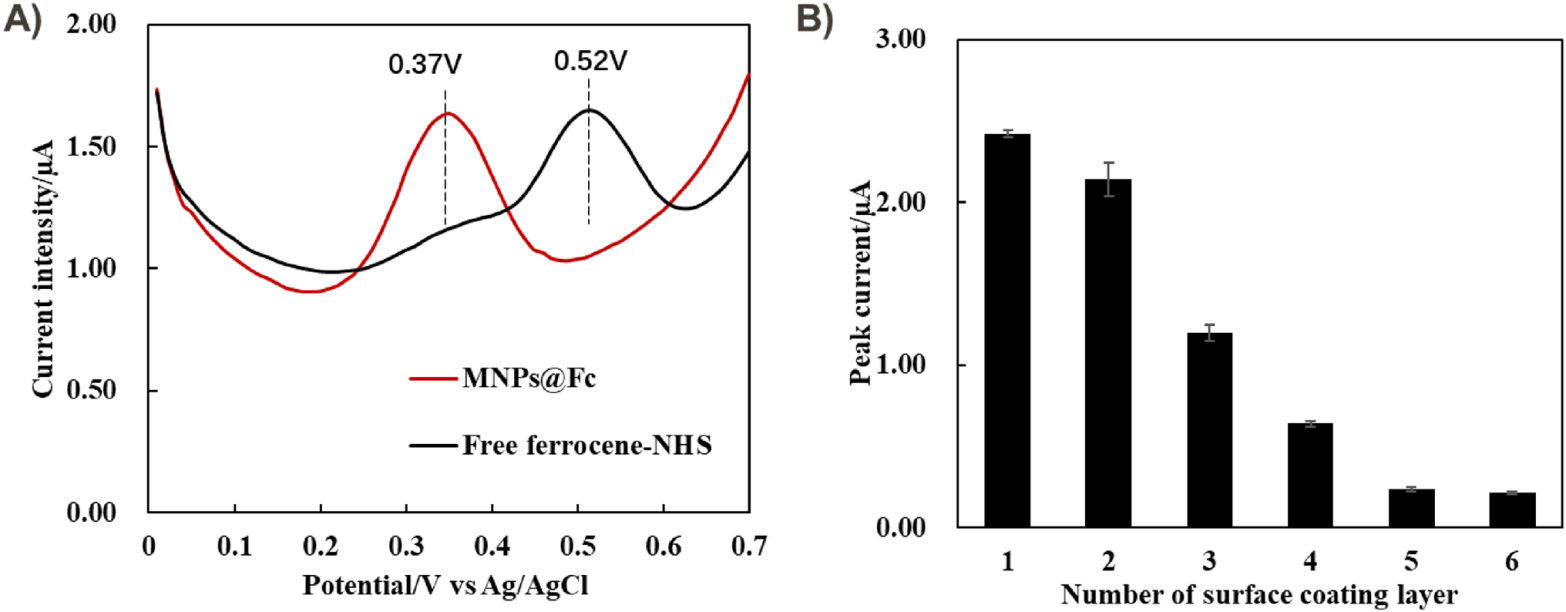
Electrochemical characterization of MNPs@Fc after the layer-by-layer coating process. **A** SWV curves of free ferrocene-NHS probes before and after conjugation; **B** Effect of surface coating polyelectrolytes layers on the peak current intensity of MNPs@Fc. The MNPs concentration was estimated to be 2.0 mg mL^−1^ in PBS1X with testing volume of 200.0 µL

In addition, the redox property of MNP@Fc after PSS and PAH coating were electrochemically characterized using SWV to assess the impact of LbL method on their redox signals [17]. Figure 4A initially compared the oxidation peak of Fc moieties before and after conjugation onto the MNP surface, where the oxidation peak of Fc was significantly shifted from 0.52 to 0.37 V. This phenomenon was assigned to the impact of side group electron withdrawing properties on the Fc moiety [46]. Due to the Fc conjugation, the amide connected to the cyclopentadienyl ring through the nitrogen instead of the carbonyl, as the existence of NHS ester for Fc moieties, which was attributed to the weaker electron withdrawing power of the nitrogen-ligated arrangement [47]. This results provided an evidence that the redox probe of Fc was covalently anchored onto the MNP surface.

After grafting each polyelectrolyte layer, MNPs@Fc was further characterized by SWV to determine their electrochemical signal intensities. The MNPs@Fc dispersed solution were directly used for electrochemical characterization in this study, because the small size of redox MNPs (~ 10 nm) was difficult to be attracted onto the microelectrode surface under a magnet within a few hours. And electrochemical measurement by using solution was dependent on the interaction of redox MNPs onto microelectrode surface via the Brownian motion [48–50]. However, from Fig. 4B (Fig. S5A), the redox intensities of MNP@Fc were decreased with the increasing number of polyelectrolyte layers on the MNP surface. There were two reasons investigated here for this: (1) These redox probes might be leaking from the MNP surface through sonication process, because the sonication of MNPs was critical to well disperse them in aqueous after centrifuge collection (CC); (2) The polyelectrolyte layer located at the MNP surface might also cover the redox probe exposure site and insulate their surface, which weakened the electron transfer efficiency of redox process for electrochemical readout.

**Fig. 5:**
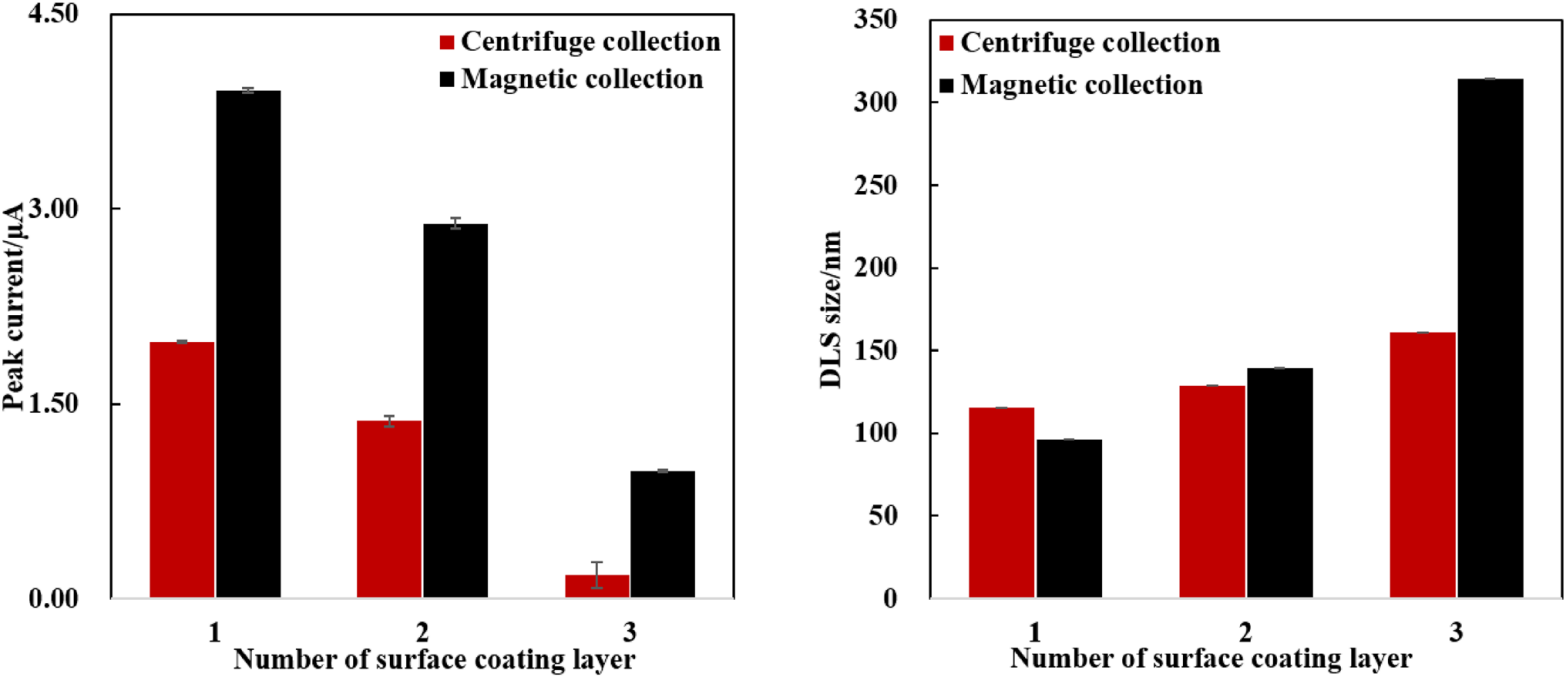
Comparison of centrifuge and magnetic collection of MNPs@Fc on the peak current intensity **A** and DLS size **B** of MNPs@Fc, during the layer-by-layer coating process. The MNPs concentration was estimated to be 1.0 mg mL^−1^ in PBS1X with testing volume of 200.0 µL

To gain insight into these issues, we choose the LS column for the efficient magnetic collection (MC) of MNPs. Compared to the CC, the MC eliminated the dependence of sonication on MNPs redispersion, thereby avoiding the loss of redox probes caused by their surface leakage. Indeed, Fig. 5A showed that the MC method indeed obtained a higher redox signal of MNPs, compared to that of CC, which was attributed to the minimal leakage of redox probes from the MNP surface. However, with the MC method, the redox intensity of the MNPs also decreased with an increase in polyelectrolyte layers. Thus, we believed that the increase in the number of polyelectrolyte coating layers might be a dominated reason for this phenomenon, since both of MC and CC method tend to a decrease of redox intensity. One possible explanation was that these polyelectrolytes hindered the exposure of the redox probes, leading to a decrease in redox signal amplification. Meanwhile, the reduction in redox intensities caused by the increase in polyelectrolyte layers can be attributed to the insulating properties of the multilayer polyelectrolyte coating.

We further characterized the DLS size of MNPs collected from both of MC and CC approaches in Fig. 5B. After grafting three polyelectrolyte layers, the MNPs collected via the MC approach appeared to be aggregate, resulting in a large DLS size of up to 314.8 ± 0.5 nm. This might be due to the presence of both hard and soft adsorption of polyelectrolyte layers during the LbL coating process. Softly adsorbed polyelectrolyte layers may be easily released from MNPs surfaces when they were dispersed in a high-ionic-strength solution [51, 52]. But sonication based CC method, as the optimized solution, can help to remove the soft adsorbed polyelectrolyte layer from the MNP surface, allowing the formation of dense and stable multi-layers.

### Three types of redox MNPs prepared by the LbL method

In addition to MNPs@Fc, this strategy was also used to construct other types of redox MNPs, by using the additional two redox probes of AQ and MB. Both of zeta potential and DLS size of MNP@AQ and MNP@MB were recorded before and after the LbL method, in Fig. S4A. Similar to the surface charge of MNP@Fc, both the AQ- and MB-conjugated MNPs still presented a positive surface in DI water, suitable for starting the LbL process. After coating with three polyelectrolyte layers of PSS/PAH/PSS, the zeta potential of these redox-active MNPs was negatively charged in Fig. S4A, while their DLS size (Fig. S4B) exhibited an stable dispersion in PBS1X buffer in Fig. S4C. From Table 1, the prepared redox MNPs with three polyelectrolyte layer coatings, presented their DLS size to be 177.3 ± 3.3 nm for MNP@Fc, 176.4 ± 0.3 nm for MNP@AQ, and 169.2 ± 2.3 nm for MNP@MB, respectively.

**Table 1:** Redox intensity, TEM and DLS size of MNPs@Fc, MNPs@AQ and MNPs@MB after LbL coating three polyelectrolytes layers

Samples	Redox intensity/µA	TEM size/nm	DLS size/nm
MNPs@Fc	0.64 ± 0.10	11.0 ± 2.0	177.3 ± 3.3
MNPs@AQ	23.25 ± 0.73	10.5 ± 2.1	176.4 ± 0.3
MNPs@MB	0.48 ± 0.13	12.4 ± 2.2	169.2 ± 2.3

^*^MNPs were dispersed in PBS1X for electrochemical measurement and DLS measurement

Figure S6A presented the sample image of the MNPs@Fc, MNPs@AQ and MNPs@MB, respectively. And from the Fig. S6B and C, the redox properties of MNPs@AQ and MNPs@MB were electrochemically characterized by SWV, where the oxidation peaks from SWV curves observed at −0.71 V and −0.34 V were assigned to the moieties of AQ and MB anchored at the MNP surface, respectively. The difference in the oxidation peak position was relevant to the intrinsic redox probes, allowing for achieving multiple detection by using two or three redox MNPs. Additionally, Table 1 summarized the redox intensity of such three redox-active MNPs, such as 0.64 ± 0.10 µA for MNP@Fc, 23.25 ± 0.73 µA for MNP@AQ and 0.48 ± 0.13 µA for MNP@MB at the MNP concentration of 1.0 mg mL^−1^. Owing to a high efficiency of redox transfer with two electrons for AQ, MNPs@AQ exhibited strongest redox intensity [53], in comparison to that of MNPs@Fc and MNPs@MB. Meanwhile, three redox MNPs (Fc, AQ, MB) were also characterized by CV, as the results displayed in Fig. S6D, E and F, respectively.

**Fig. 6:**
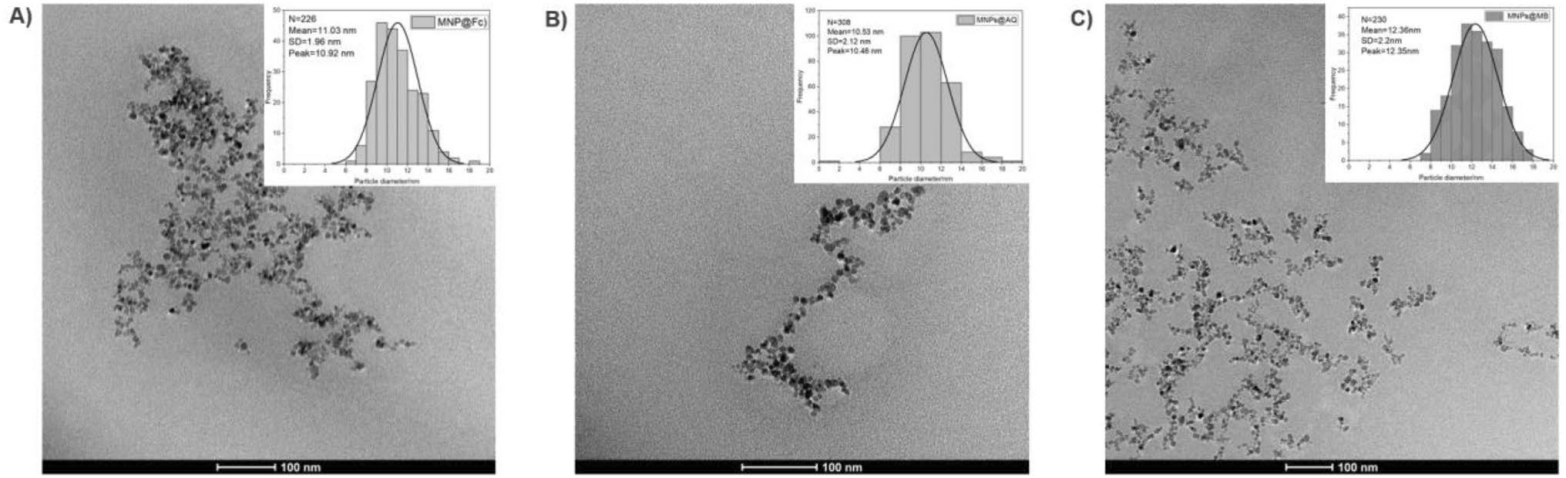
TEM images and histograms of particle size distribution for MNPs@Fc **A**, MNPs@AQ **B** and MNPs@MB **C**, after coating three polyelectrolytes layers by LbL method

To clarify that the increase of DLS size was dominated by the LbL method, rather than the MNP aggregation, we measured the TEM image of MNPs after preparation, which displayed the physical structure of MNPs after LbL surface coating. Figure 6 showed the TEM micrographs for three kinds of MNPs, where their average particle sizes were determined from the histograms to be 11.0 ± 2.0 nm for MNP@Fc, 10.5 ± 2.1 nm for MNP@AQ, and 12.4 ± 2.2 nm for MNP@MB, as summarized in Table 1. Through these TEM micrographs, there were no obvious increase of TEM size, compared to that of initial iron oxide nanoparticle with approximately TEM size of 8.7 nm [12, 54]*.* Thus, the redox conjugation and the LbL surface coating process contributed less to MNP aggregation in our study, while the main reason for their increase of DLS size was mainly caused by the polyelectrolyte shells that changed the surface properties of MNPs.

### Construction of IgG based bio-surface

**Fig. 7:**
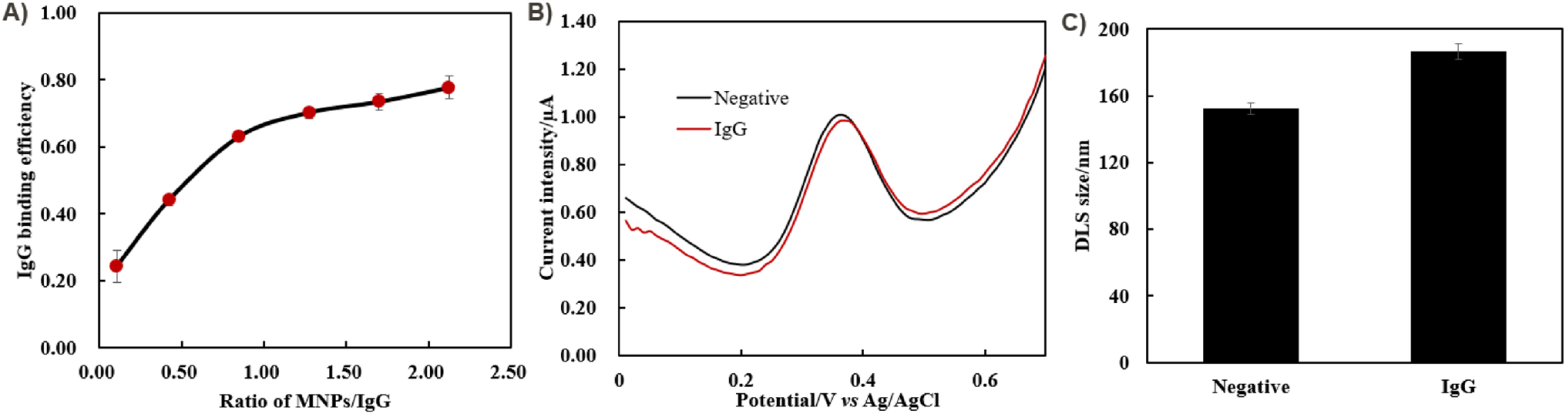
Effect of IgG attachment on the redox-MNPs surface. **A** Binding efficiency of IgG onto MNPs@Fc surface under the ratio of MNP/IgG from 0 to 2.12. **B** SWV curves of MNPs@Fc with or without IgG conjugation. (1.0 mg mL^−1^ in 200.0 µL of PBS1X). **C** The DLS size of MNPs with or without IgG conjugation in PBS1X

Towards to the application of electrochemical sensor, it was critical to construct a bio-probe onto the MNP surface, so that they can be used for bio-capture. Thus, we selected the IgG as a model of bio-probe for the development of multi-functionality onto MNPs, because the IgG was the most common type of antibody, involving two heavy chains and two light chains. The MNPs used for IgG construction have been grafted with three polyelectrolyte layers of PSS/PAH/PSS, where the outside layer of MNPs was terminated with negatively charged PSS. Considering the isoelectric point of IgG was around pH 6.8 and it had a negative net charge at a pH of 7.4, the hydrophobicity of the negatively charged PSS played the key role to drive the adsorption of the IgG onto their surface, via the hydrophobic forces predominantly process [30, 55, 56]. Meanwhile, their adsorption attraction between positively charged segments on IgG and negatively charged PSS were probably also contributing to form a corona of IgG at the MNP surface [29]. By SDS-PAGE gel analysis, the interaction between IgG and MNPs have been investigated to confirm the IgG binding efficiency, under the range of the MNPs/IgG ratio from 0 to 2.12. As depicted in Fig. S7A, we observed that the gel bands of IgG was located around 150 kDa, following Coomassie blue staining of the gel image. While free IgG migrated through the SDS-PAGE gel, IgG bound to the MNPs suffered from hindered migration, leading to a reduction in detectable IgG. Thus, the binding efficiency of IgG on the MNP surface was assessed in Fig. 7A, where an increase of MNPs/IgG ratio retained more IgG at MNPs surface, with a high IgG binding efficiency. Typically, the IgG binding efficiency significantly increased from 24 to 63% when the MNP/IgG ratio was raised from 0.11 to 0.85. When the ratio was further increased from 0.85 to 2.12, the binding efficiency showed merely a slight increase, from 63 to 78%.

To confirm the successful incorporation of multi-functionality in these MNPs, their redox properties were further characterized by SWV following IgG binding. In Fig. 7B, the characteristic redox peak of MNP@Fc was observed at 0.37 V, with or without IgG binding. From Fig. S7B, MNP@Fc and IgG-bound MNPs@Fc showed their electrochemical response of approximately 0.52 ± 0.02 µA and 0.51 ± 0.01 µA, respectively. Such similar redox intensities from both samples indicated that the IgG binding process had a minimal effect on the redox properties of the MNPs. Additionally, we measured the DLS size of the MNPs before and after IgG binding to evaluate their stability. As shown in Fig. 7C, the DLS size of the MNPs before and after IgG binding were determined to be 152.3 ± 3.3 nm and 186.5 ± 4.8 nm, respectively. Although there was a slight increase in DLS size owing to the presence of IgG, the MNP stability in PBS buffer was confirmed, highlighting their successful preparation with magnetic, redox, and biological properties.

## Conclusions

Surface modification strategies were a critical step from nanoparticle synthesis to their broad application. Surface modification of MNPs not only enhanced their dispersion stability by addressing challenges of physicochemical instability and poor biocompatibility but also expanded their potential applications. Due to various functional groups, distinct charge densities, and hydrophobic properties, the use of organic solvents was necessary for the surface modification processes of MNPs. However, our approach of LbL coating was compatible to transfer these MNPs from the organic solvent to an aqueous solution, which maintained their stability in high ionic strength buffers and prevented their aggregation in the aqueous solution. Meanwhile, the polyelectrolyte shell offered an desirable interface to form a biomolecules corona without the needs of further chemical crosslinking. Our study emphasized the effectiveness of the LbL coating technique for constructing multifunctional MNPs, while also mitigating the risk of their aggregation.

Toward to the field of electrochemical sensors, we developed multifunctional ultra-small nanoparticles with magnetic, redox, and biological properties using a facile LbL coating strategy. Firstly, the ultra-small size of MNPs@Fc, MNPs@AQ and MNPs@MB were measured using TEM to be11.0 ± 2.0 nm, 10.5 ± 2.1 nm, and 12.4 ± 2.2 nm, respectively. Secondly, as an examination of the redox signal amplification from developed MNPs, the redox signal of MNPs@Fc, MNPs@AQ and MNPs@MB were successfully characterized by SWV, with their redox intensity of 0.64 ± 0.10 µA, 23.25 ± 0.73 µA and 0.48 ± 0.13 µA, respectively. Thirdly, the IgG as the bio-probe model were also used to construct the bio-property at MNP surface, where the binding efficiency of IgG onto the PSS terminated MNPs was estimated to be 78% via the SDS-PAGES. 

Our approach demonstrated the successful integration of redox moieties for signal amplification and bio-probes for target recognition onto these MNPs, offering immense potential for the disease biomarker diagnosis and detection. These advancements underscore the feasibility of MNP-derived electrochemical biosensors and their applicability in point-of-care diagnostic assays.

## Supplementary Information

Supplementary Material 1.

Supplementary Material 2.

## Data Availability

Data is provided within the manuscript or supplementary information files and all the data will be made available for the request.
