# Supplementary figures and images for: Layer-by-layer coating strategy to functionalize the magnetic nanoparticles for their multi-functionalization
Source: Discov Nano. 2025 May 2;20(1):74. doi: 10.1186/s11671-025-04250-6 (PMC12048377; doi:10.1186/s11671-025-04250-6)

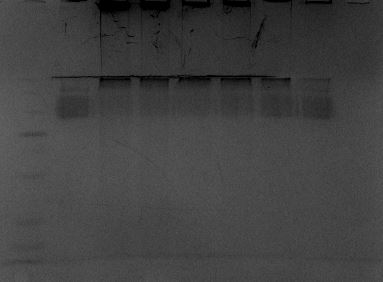

Supplement: Supplementary file 1 — Supplementary Material 1. [file 11671_2025_4250_MOESM1_ESM.jpg]
