# Supplementary material for: Layer-by-layer coating strategy to functionalize the magnetic nanoparticles for their multi-functionalization
Source: Discov Nano. 2025 May 2;20(1):74. doi: 10.1186/s11671-025-04250-6 (PMC12048377; doi:10.1186/s11671-025-04250-6)
Supplement: Supplementary file 2 — Supplementary Material 2. [file 11671_2025_4250_MOESM2_ESM.docx]

**Supplementary materials**


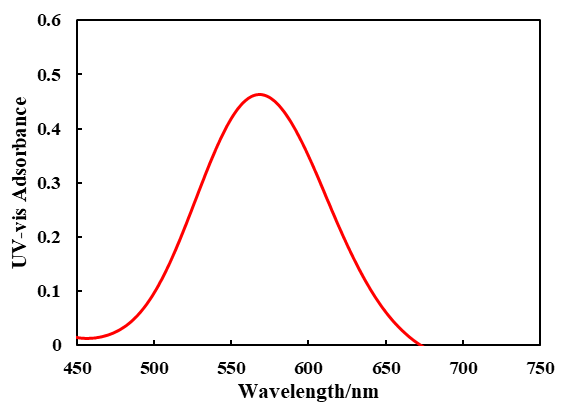

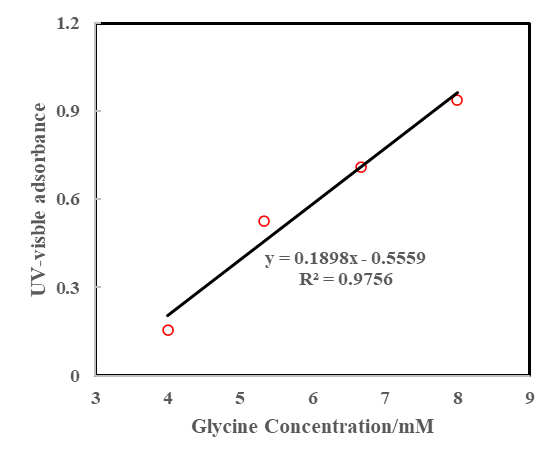


**Figure S1.** Ninhydrin testing to quantify the amino groups density for these MNPs@NH_2_. **A)** UV-visible spectra of Ninhydrin and **B)** Calibration curve from glycine sample.

**Calculation method for amino density:**

In our study, the synthesis of iron oxide are Fe_2_O_3_ with their density of iron(III) oxide (5.2 g.cm^-3^) and the physical size of Fe_2_O_3_ was estimated to be 10 nm for subsequent calculation.

On one side, we will calculate the weight (2.72×10^-15^mg), volume (523nm^3^), and surface area(314 nm^2^) of single iron oxide, according to the diameter and density of Fe_2_O_3_. Therefore, according to the volume (200µL) and concentration (5.95mg.mL^-1^) of the sample solution (iron oxide solution), we can calculate the total surface area of nanoparticle used in the testing.

On other side, from the Ninhydrin testing, we have measured the UV-visible spectra(Figure S1A) and established the calibration curves(Figure S1B), where the glycine was used as the amino-group for measurement. In our study, the glycine was replaced by the solution of MNPs@NH_2_ to measure the amino groups, using the same protocol. According to the UV-visible absorbance and calibration curve, we can calculate the equal concentration and mole of glycine(amino density). It equals to the total number of amino groups from the MNPs@NH_2_. In summary, the calculated the total number of the amino groups was divided by the total surface area of nanoparticle, to achieve the NH_2_ density per nm^2^ for the different sample.


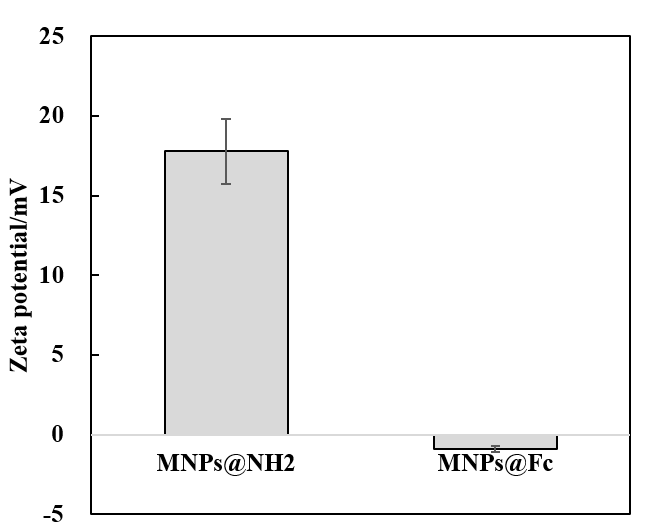


**Figure S2.** Zeta potential of MNPs@NH_2_ and MNPs@Fc in 10.0 mM HEPES with pH=7.4


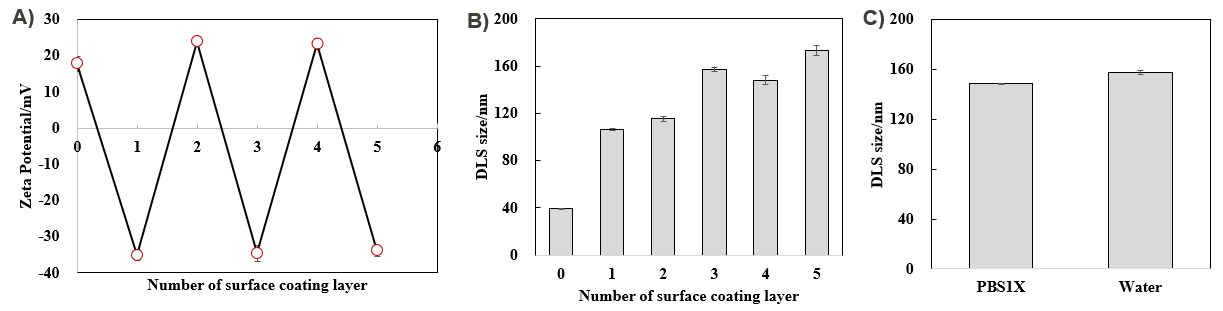


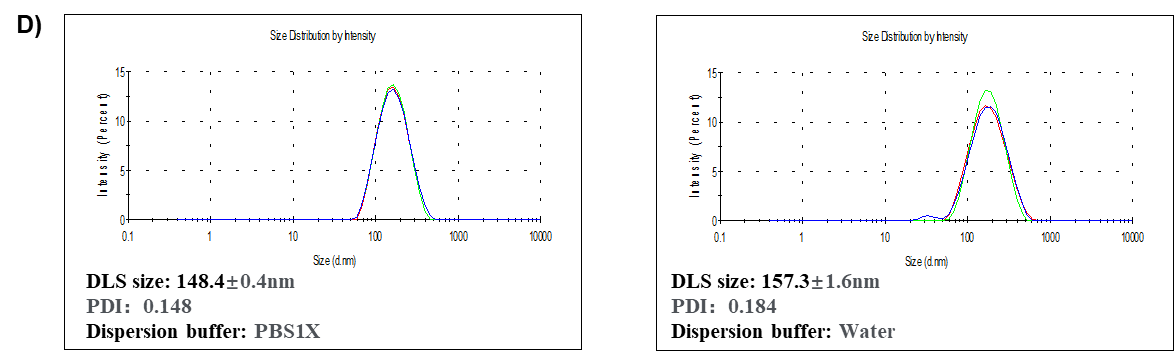


**Figure S3. A)** Zeta potential shifting phenomena of MNPs@NH_2_ caused by the coating polymer of positively charged PSS and negatively charged PAH under LBL method(measured in 10 mM HEPES, pH=7.4) **B)** DLS size of MNPs@NH_2_ coated with different number of layers of PSS or PAH, measured in water. **C)** DLS size stability of MNPs@NH_2_ with three layer of PSS/PAH/PSS once redispersed in PBS1X and water. **D)** Raw data of DLS measured in both of water and PBS1X for the sample of MNPs@NH2 after 3 layers of LBL coating.


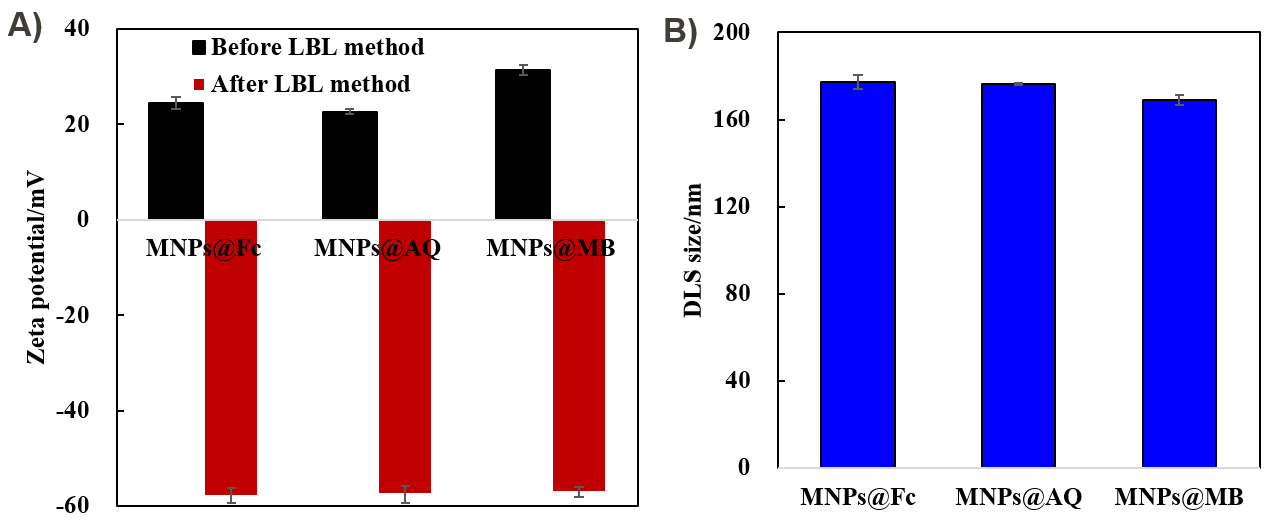


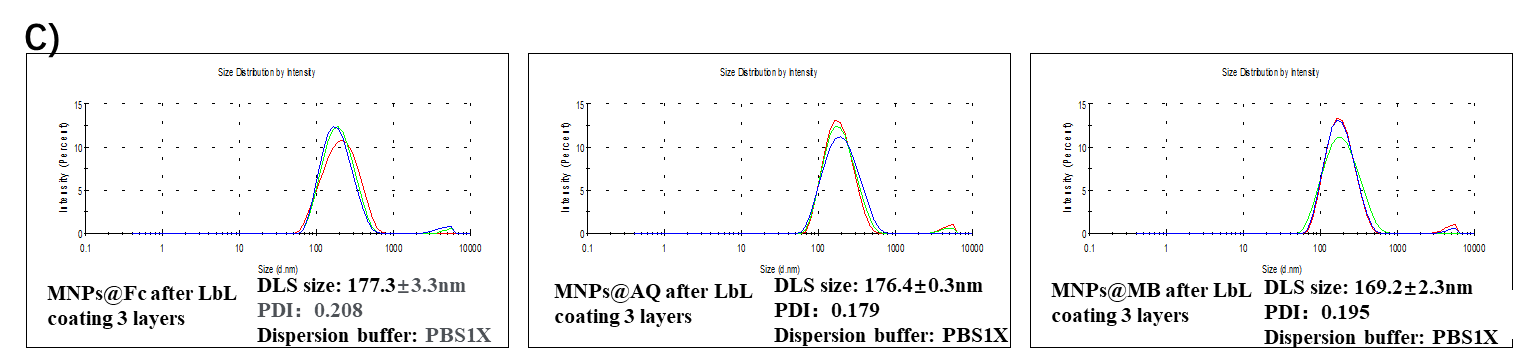


**Figure S4. A**) Zeta potential of three redox MNPs before and after LBL method, measured in water. **B**) DLS size of MNPs after LBL in PBS1X. **C)** Raw data of DLS measured in PBS1X for the three kinds of redox MNPs after 3 layers of LBL coating.


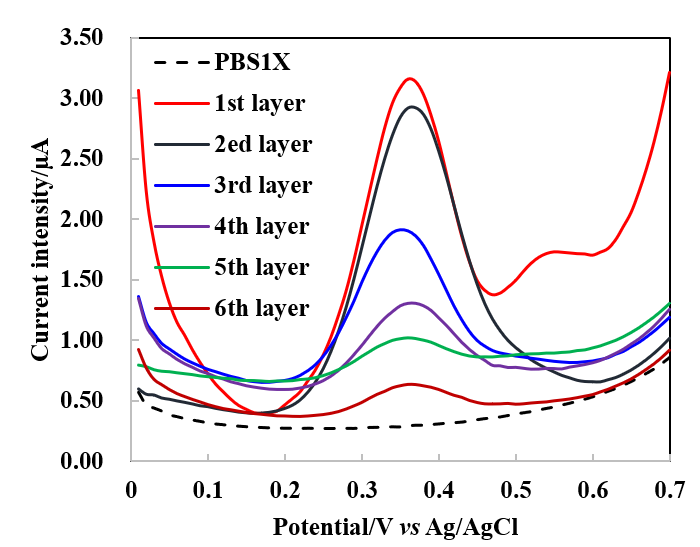


**Figure S5.** SWV curves of MNPs@Fc coated with multi-polyelectrolytes layers.


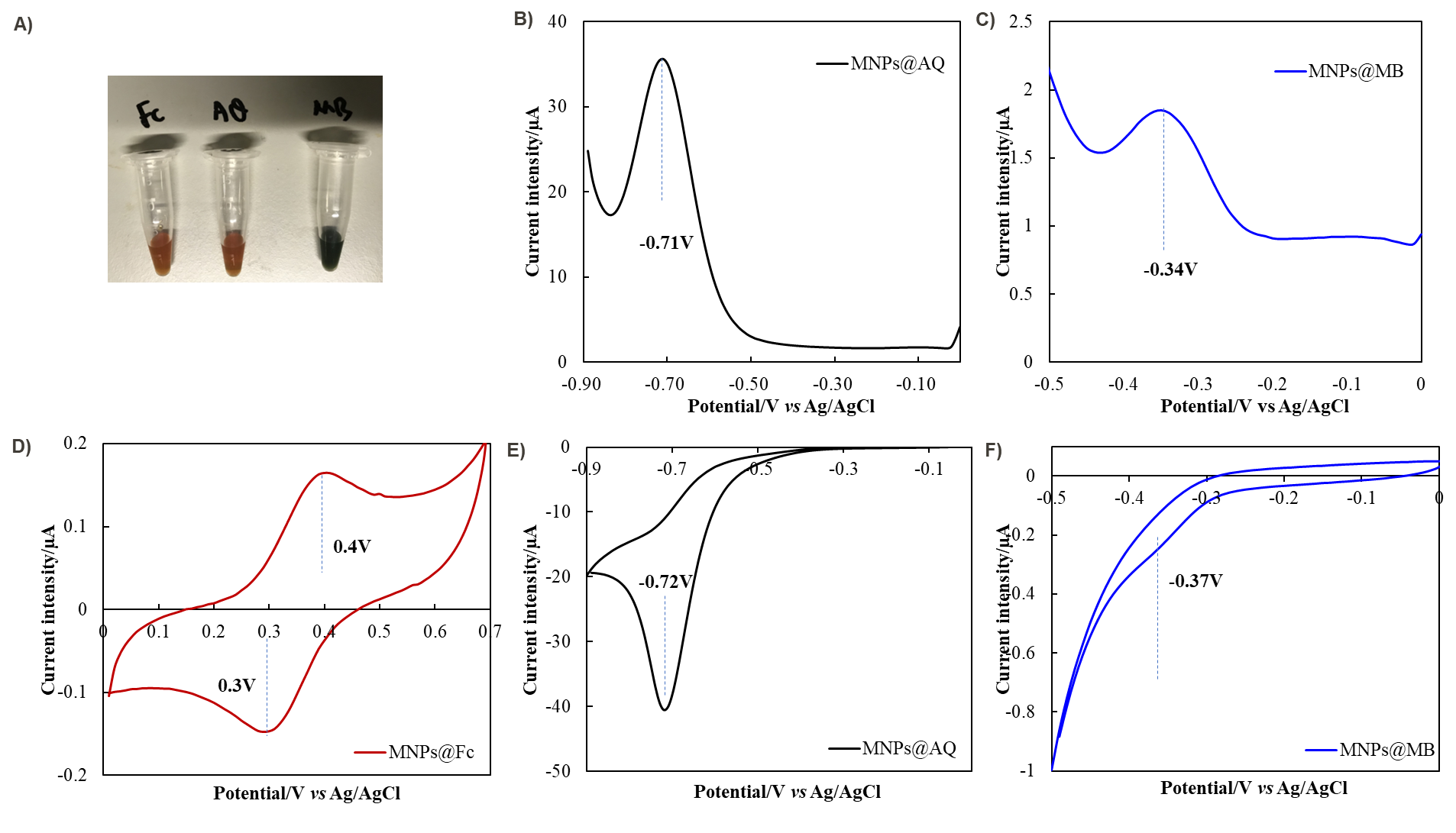


**Figure S6. A)** Images of prepared MNPs@Fc, MNPs@AQ and MNPs@MB. CV and SWV curves of MNPs@AQ (**B, E**), MNPs@MB(**C,F**) and MNPs@Fc(**D**) after coating three polyelectrolytes layers. The MNPs concentration was 1.0 mg.mL^-1^ in PBS1X with testing volume of 200.0 µL.


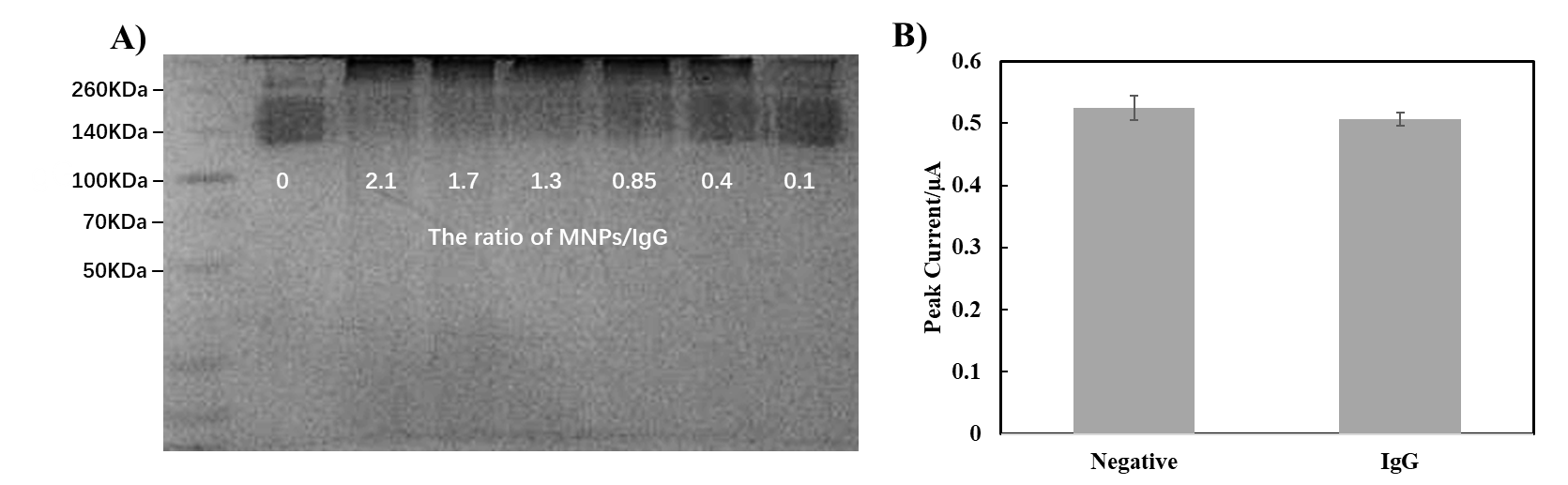


**Figure S7. A)** SDS-page of IgG with or without bound to MNPs@Fc under the ratio of MNPs/IgG from 0 to 2.12. **B)** Peak current intensity of redox MNPs with or without IgG conjugation at the ratio of 2.1

One particle of MNPs@Fc was estimated to be the weight of 3.44×10^-15^ mg, depending on the TEM size of 10 nm. The concentration of MNPs@Fc and IgG were around 2.2 mg.mL^-1^ and 0.5 mg.mL^-1^. 3.0 µL of IgG was added inside the tube with 1.0, 4.0, 8.0, 12.0, 16.0, 20.0 µL of MNPs@Fc for the formation of corona surface. All the sample was running by SDS-PAGE gel image and the intensity of gel bands was estimated by Image J software from the SDS-PAGE of IgG adsorption. In the mixture of IgG and MNPs, these IgG were adsorbed onto MNPs surface to form a corona particle, but the free IgG remaining in the solution will go through the SDS-PAGE with detectable signal by Commaise blue staining. The equation of binding efficiency was presented in the following, according to the Gel intensity from Figure S6A:

$$The binding effciency=\frac{x\left( total IgG \right)-x(free IgG)}{x(total IgG)}$$
